# Evaluating Behavioral Impacts of School Cancer Education Programs Several Months and Years After Implementation in Japan

**DOI:** 10.1007/s13187-025-02655-6

**Published:** 2025-05-24

**Authors:** Masanari Minamitani, Atsuto Katano, Shingo Ohira, Takuya Hayashi, Keiichi Nakagawa

**Affiliations:** 1 Department of Radiology, The University of Tokyo Hospital, 7-3-1 Hongo, Bunkyo-Ku, Tokyo, 113-8655 Japan; 2 Department of Comprehensive Radiation Oncology, The University of Tokyo, 7-3-1 Hongo, Bunkyo-Ku, Tokyo, 113-0033 Japan; 3 Department of Radiological Science, Graduate School of Human Health Science, Tokyo Metropolitan University, 7-2-10 Higashi-Ogu, Arakawa-Ku, Tokyo, 116-8551 Japan

**Keywords:** School health education, Cancer education, HPV vaccination, Behavioral change, Japan

## Abstract

School-based cancer education programs have recently been introduced in Japan to promote cancer awareness and encourage students to value their health and lives. Although short-term behavioral effects have been reported, their long-term effects remain unclear. We evaluated the mid- and long-term effects of a cancer education program conducted in rural Japanese high schools between 2016 and 2019. The program featured lectures by an oncologist and a cancer survivor. In total, 741 former students from four participating schools were invited to complete an online survey. Participants were grouped by their memory of the program: “no memory of the program,” “no memory of the contents,” and “memory of the contents.” Data on human papillomavirus (HPV) vaccination during school days, cervical cancer screening, intention to undergo cancer screening, and health literacy were collected and analyzed. Valid responses were obtained from 96 participants with an average age of 24.1 years. Female participants with a vivid memory of the program were significantly more likely to have received the HPV vaccine (100%) than those without such a memory (63.5%, *p* = 0.047). However, no significant associations were found between memory of the program and cervical cancer screening behavior or future screening intentions. School-based cancer education may influence HPV vaccination behavior on a several-month timescale but appears to have limited effects on long-term cancer prevention behavior. The program content may need to be optimized to enhance medium-term behavioral changes.

**Supplementary Information:**

The online version contains supplementary material available at 10.1007/s13187-025-02655-6.

## Background

Recently, cancer education programs have been introduced as a part of health education in Japanese schools [1]. Since 1981, cancer has been the leading cause of death in Japan, making it a public health priority [2]. The primary goals of school-based cancer education programs are to help children gain accurate knowledge about cancer and encourage them to reflect on the importance of health and life [3].

A key feature of these programs is the inclusion of visiting lecturers, either healthcare professionals working in oncology or cancer survivors, who are invited to the school to deliver educational sessions [3]. However, the implementation of these visiting lecturer-based programs has progressed slowly. Commonly cited barriers include a lack of time within the school curriculum, a shortage of suitable lecturers, and insufficient compensation for the efforts of the lecturers.

Previous studies have assessed the effectiveness of health education programs in schools [4–7]. From a short-term perspective, students’ knowledge of cancer improved significantly immediately after the program [4]. In the medium-term evaluation, temporary improvements were observed in both knowledge and attitudes toward cancer prevention; however, these effects often weakened several months after the intervention [5, 6]. Our previous study evaluated a cancer education program in Japan but did not find statistically significant long-term effects approximately ten years after the program [7]. However, that study was limited to a single private all-boys school in Tokyo [7]. To the best of our knowledge, no other studies have examined the long-term effects of school-based health education, and thus, much remains unknown.

This study aimed to evaluate the mid- and long-term effectiveness of cancer education programs targeting male and female students in public schools located in a rural area. By demonstrating the benefits of such programs in a broader and more representative setting, we hope to contribute to the promotion and enhancement of cancer education in Japan.

## Methods

The *Kochiken-sogo-hoken-kyokai* (Kochi Comprehensive Health Association) is a public interest foundation located in Kochi Prefecture, a rural area in Japan (https://www.hokyo.or.jp/). The association works to improve resident health through health checkups and health promotion activities. Since 2014, the association has been delivering cancer education programs, which involved both an oncologist and a cancer survivor as visiting lecturers, to local high schools.

The program included the following components:

- A 30-min lecture on basic cancer knowledge by an oncologist
- A 45-min talk by a cancer survivor sharing his/her experience
- A 60-min group discussion focused on common misconceptions about cancer, the need for cancer education, and ways to help prevent cancer among families.
- A 10-min explanation of the local government’s cancer control initiatives

These programs were provided free of charge to the schools.

We invited 12 high schools that implemented the program to participate in a follow-up survey of their graduates. With the assistance of the Kochi Comprehensive Health Association, four schools, School A, B, C, and D, agreed to cooperate (Supplementary Table 1). In all the four high schools, the same oncologist (a radiation oncologist) and cervical cancer survivor were involved in program delivery.

Our study targeted graduates from the relevant grades at these four schools. In total, 741 individuals were eligible to participate. The survey design was based largely on a previous study and included items on participant characteristics (sex, age, residence, employment status, educational background, medical history, health literacy, and five health-related behaviors: smoking, alcohol consumption, diet, physical activity, and body shape perception) [7]. Other items included willingness to undergo cancer screening in the future (measured on a 5-point Likert scale) and memory of the cancer education program. For only female participants, human papillomavirus (HPV) vaccination status during school days and history of cervical cancer screening, which Japanese women are recommended to undergo from the age of 20 every two years by cytology testing, were included [8]. To assess health literacy, we used the Japanese translation of the short version of the European Health Literacy Survey Questionnaire (HLS-Q12) [9, 10]. This scale yielded a total score ranging from 0 to 50. Participants who answered “don’t know” to more than 20% of the items were excluded from the analysis.

Participating schools sent invitation letters to eligible graduates, which included information about the study and a URL linked to the online survey. After providing informed consent, the participants accessed the website and completed a questionnaire comprising 30–35 items. Those who completed the survey received a reward worth JPY 500.

Participants were classified into three groups based on their memory of the cancer education program: (1) “No memory of the program,” (2) “No memory of the contents,” and (3) “Memory of the contents.” We compared the participant characteristics across these groups. We used the chi-square test for categorical variables and analysis of variance (ANOVA) for continuous variables to compare the three groups. For female participants, we used the Fisher exact test to compare the HPV vaccination status between “No memory of the program and the contents” and “Memory of the contents.” All statistical analyses were conducted using the SPSS version 27, with a significance level set at 5%.

## Results

The participating schools began sending invitation letters on November 13, 2024, and the survey was closed on January 31, 2025. Of the 741 eligible candidates, 114 (15.4%) completed the survey during the study period. Eighteen participants were excluded due to invalid responses on the HLS-Q12 (e.g., more than 20% “don’t know” responses), resulting in 96 valid responses for analysis. Fourteen participants did not claim rewards, despite being eligible.

The participant characteristics are shown in Table 1. Totally 25 men and 71 women completed the survey. The average age was 24.1 years. Regarding memory of the cancer education program, 38 participants (39.6%) were classified as having “No memory of the program;” 49 (51.0%), “No memory of the contents;” and 9 (9.4%), “Memory of the contents.” A higher proportion of women was observed in the group with a clearer memory of the program, without a significant difference. Additionally, 28.1% of the participants had an educational background in the medical and welfare fields. Two women had a history of cancer experience, each of whom were classified into “No memory of the program” and “Memory of the contents.” Demographic characteristics or health-related behaviors did not differ significantly among the groups.

**Table 1:** Characteristics of survey participants

	No memory of the program		No memory of the contents		Memory of the contents		
	(*N* = 38)		(*N* = 49)		(*N* = 9)		
	N	%	N	%	N	%	*p*-value
Sex							0.26
Male	13	34.2%	11	22.4%	1	11.1%	
Female	25	65.8%	38	77.6%	8	88.9%	
Age							0.41
Mean, (SD)	24.2	1.08	24.0	1.06	24.4	1.33	
Marriage							0.27
Single/divorced	36	94.7%	43	87.8%	7	77.8%	
Married	2	5.3%	6	12.2%	2	22.2%	
Work status							0.086
Not working	6	15.8%	7	14.3%	4	44.4%	
Worker	32	84.2%	42	85.7%	5	55.6%	
Last or current educational background							0.34
Medicine & welfare	8	21.1%	17	34.7%	2	22.2%	
Other	30	78.9%	32	65.3%	7	77.8%	
Past medical history							0.86
None	26	68.4%	34	69.4%	7	77.8%	
Any	12	31.6%	15	30.6%	2	22.2%	
Family cancer history							0.73
None	16	42.1%	23	46.9%	3	33.3%	
Any	22	57.9%	26	53.1%	6	66.7%	
Smoking							0.51
Recommended	33	86.8%	44	89.8%	9	100%	
Non-recommended	5	13.2%	5	10.2%	0	0%	
Alcohol							0.78
Recommended	36	94.7%	47	95.9%	9	100%	
Non-recommended	2	5.3%	2	4.1%	0	0%	
Exercise							0.11
Recommended	11	28.9%	17	34.7%	0	0%	
Non-recommended	27	71.1%	32	65.3%	9	100%	
Salt meal							0.42
Recommended	36	94.7%	48	98.0%	8	88.9%	
Non-recommended	2	5.3%	1	2.0%	1	11.1%	
Obesity							0.59
Recommended	25	65.8%	27	55.1%	5	55.6%	
Non-recommended	13	34.2%	22	44.9%	4	44.4%	
HLS- Q12 score							0.75
Mean, (SD)	26.4	8.9	27.7	7.8	26.4	7.1	

*Abbreviation*: HLS- Q12: a short version of the European Health Literacy Survey Questionnaire (HLS-EU-Q47)

Recommendation cut-off points: never smoked (smoking), weekly alcohol consumption of < 150 g (alcohol),: ≥ 37.5 and ≥ 31.9 metabolic equivalent hours per day for male and female (exercise), consumption of < 0.67 g of fish roe per day (salt meal), body mass index within the range of 21–27 for male and 19–25 for female (obesity)

The results for female participants are shown in Table 2. The HPV vaccination status did not differ across the three groups overall. However, on comparing those with “No memory of the program or contents” to those with “Memory of the contents,” all participants in the latter group had received the HPV vaccine, representing a significantly higher proportion (63.5% vs. 100%, *p* = 0.047). Cervical cancer screening history did not differ among the groups.

**Table 2:** Relationship between memory of the program and preventive behaviors among female participants

	No memory of the program		No memory of the contents		Memory of the contents		
	(*N* = 25)		(*N* = 38)		(*N* = 8)		
	N	%	N	%	N	%	*p*-value
HPV vaccination during school days							0.095
Vaccinated	17	68.0%	23	60.5%	8	100%	
Not vaccinated	8	32.0%	15	39.5%	0	0%	
Cervical cancer screening							0.937
Recommended	8	32.0%	14	36.8%	2	25.0%	
Experienced but not follow to recommendation	10	40.0%	12	31.6%	3	37.5%	
Never experienced	7	28.0%	12	31.6%	3	37.5%	

*Abbreviation*: HPV: human papillomavirus

Table 3 presents the participants’ willingness to undergo cancer screening in the future, based on a 5-point Likert scale. No significant differences were observed across the three groups based on memory.

**Table 3:** Relationship between memory of the program and intention to undergo cancer screening

	No memory of the program		No memory of the contents		Memory of the contents		
	(*N* = 25)		(*N* = 38)		(*N* = 8)		
	N	%	N	%	N	%	*p*-value
Intention to undergo cancer screening							0.95
Very high	16	42.1%	18	36.7%	3	33.3%	
Slightly high	15	39.5%	21	42.9%	5	55.6%	
Neutral	5	13.2%	9	18.4%	1	11.1%	
Slightly low	2	5.3%	1	2.0%	0	0%	
Very low	0	0%	0	0%	0	0%	

## Discussion

This study investigated the effects of school-based cancer education programs on participants’ health-related behaviors by comparing outcomes based on their degree of memory of the program. Female participants were more likely to recall the program content clearly as compared to male participants, and those with vivid memories were more likely to have received the HPV vaccine. In contrast, memory of the program was not associated with other current or future health behaviors, such as lifestyle habits, history of cervical cancer screening, or intention to undergo cancer screening in the future.

Memory of the cancer education program and HPV vaccination status were significantly associated. In Japan, the government began recommending HPV vaccination for girls aged 12–16 years in 2010 and free vaccination started in 2013 [11]. However, shortly thereafter, the recommendation was suspended following sensationalized media coverage of the alleged adverse effects and was not reinstated until 2021 [11]. To address the nine-year gap in HPV vaccination suspension, the government conducted a catch-up vaccination program from April 2022 to March 2025, targeting women born on from 1997–2008 and unvaccinated with HPV [12]. The participants in this study were born in this period. According to a recent report by the Ministry of Health, Labour and Welfare, HPV vaccination coverage, including catch-up vaccinations, among individuals born between April 2000 and March 2003 (a group with historically low uptake) ranged from 34.5% to 40.6% [13, 14]. In contrast, in our study, the HPV vaccination rate among participants with “No memory of the program or contents” ranged from 60.5% to 68.0%, whereas it was 100% among those with “Memory of the contents.” Because most participants were within the target age for HPV vaccination during the program, despite the national recommendation being suspended at the time, the cancer education program may have influenced their decision to be vaccinated. This suggests a potential medium-term impact on health behaviors on a scale of several months.

However, memory of the program was not associated with current health behaviors or future intentions. The proportion of participants engaging in health-related behaviors was similar to that observed in our previous survey [7]. Among the female participants, 33.8% reported undergoing regular cervical cancer screening, although the rates were comparable to those reported for the same age group in Kochi Prefecture in 2022, which was 12.5% in the age group of 20–24 years and 30.0% in the age group of 25–29 years [15].

Additionally, 81% of the participants expressed an intention to undergo cancer screening in the future, which aligns with previous Japanese studies that reported rates between 68 and 91% [7, 16]. These findings suggest that, regardless of whether individuals currently remember the cancer education program several years after its implementation, such memory is not associated with actual behavioral changes. This may indicate that the program has limited long-term effects on behavioral change over a multi-year time scale.

Previous studies have reported the effectiveness of school-based health education programs, including cancer education, mainly in the short-to-medium term. For example, a Japanese study evaluating cancer prevention education in elementary schools found a significant improvement in correct responses regarding cancer risk factors one week after the program [4]. A Korean study examined the effects of cancer-prevention education on elementary school students’ self-reported knowledge, attitudes toward cancer preventability, self-efficacy, and behavioral intentions three months after the intervention [5]. The results showed partial effects; although knowledge and attitudes improved, there were no significant changes in self-efficacy or behavioral intentions [5]. Similarly, a study from the UK reviewed health education programs that covered topics such as cancer basics, warning signs, diagnosis, and treatment for adolescents aged 11 to 17 years [6]. Six months after the program, participants demonstrated improved recognition of “cancer warning signs,” but there were no significant differences in awareness regarding “the relationship between cancer and age” or knowledge of “common cancers [6]. Another Japanese study that assessed the long-term effectiveness of cancer education several years after implementation found no significant difference in the willingness to undergo cancer screening between the intervention and control groups [7]. Our findings are consistent with these previous findings, suggesting that cancer education programs may have short- to medium-term impacts, ranging from weeks to several months, but are unlikely to produce sustained behavioral changes over several years.

## Limitation

This study had several limitations. First, the eligible response rate was relatively low, at only 13.0% (96 out of 741), which limited the generalizability of the findings to the overall population of program participants. Additionally, the HPV vaccination rate among all participants was higher than that of the general population, and approximately 30% of the respondents had educational backgrounds in the medical or welfare fields, which is also much higher than the average. Although the response rate was not high, it was considered acceptable for online surveys where rates below 10% are common [17]. Second, the sample size was small, limiting the generalizability of the findings. However, these results do not necessarily reflect the overall effectiveness of cancer education programs in Japan. Third, this study employed a cross-sectional rather than longitudinal design. Although we observed a significant association between memory of the cancer education program and HPV vaccination status, we could not determine causality. It remains unclear whether participation in the program led to vaccination, whether vaccinated individuals were more likely to remember the program, or whether a third unmeasured factor influenced both. Fourth, this study compared participants based on the degree of memory of the program, rather than on whether they actually participated in the program or not. Our original research plan intended to compare program participants with a control group (i.e., graduates from the same schools who did not receive the program). However, the cooperating schools did not permit us to include non-participant graduates, leaving us with no choice but to classify participants based on their memory of the program. Despite these limitations, our findings contribute to the growing body of research on school-based health education and offer insights into improving the design and evaluation of cancer education programs.

Based on the findings of this study, we believe that it is necessary to reconsider and revise the goals of school-based cancer education programs in Japan. Unlike other high-income countries, Japan continues to face increasing cervical cancer mortality, largely because of its low HPV vaccination and screening rates [18]. Given that school health education appears to influence student behavior on a medium-term timescale, cancer education programs should be designed strategically to promote HPV vaccination, particularly among adolescent girls. Furthermore, local governments in Japan have recently begun subsidizing HPV vaccination for boys aged 12–16 years. Although this study found that female participants were more likely than male participants to remember the content of the program, future efforts should focus on making the educational experience more engaging and memorable for male students as well.

Notably, this study evaluated the effectiveness of cancer education programs implemented over five years ago on a trial basis prior to their integration into the official school curriculum. Since then, the content and guidelines of these programs have been more thoroughly developed and standardized. Updating the program’s content based on the insights gained from this study could enhance its relevance and impact on current and future students.

## Conclusion

This study demonstrated that memory of school-based cancer education programs was significantly associated with HPV vaccination status, but not with cervical cancer screening behavior or intention to undergo screening in the future. These findings suggest that school health education programs may influence adolescent health behaviors on a medium-term timescale. Therefore, the content and structure of cancer education programs should be carefully designed to maximize their impact and support public health goals.

## Supplementary Information

Below is the link to the electronic supplementary material.

Supplementary file1 Supplementary Table 1. Details of Cancer Education Programs Implemented at Participating High Schools (XLSX 11 KB)

## Data Availability

The data that support the findings of this study are available from the corresponding author, M.M., upon reasonable request.
